# Cell Cycle Dysregulation and Renal Fibrosis

**DOI:** 10.3389/fcell.2021.714320

**Published:** 2021-11-25

**Authors:** Yun-Shan Wu, Shan Liang, Dong-Yi Li, Jun-Hao Wen, Ji-Xin Tang, Hua-Feng Liu

**Affiliations:** ^1^ Key Laboratory of Prevention and Management of Chronic Kidney Disease of Zhanjiang, Institute of Nephrology, Affiliated Hospital of Guangdong Medical University, Zhanjiang, China; ^2^ Shunde Women and Children’s Hospital, Guangdong Medical University (Foshan Shunde Maternal and Child Healthcare Hospital), Foshan, China

**Keywords:** cell cycle arrest, over proliferation, cell senescence, senescence associated secretory phenotype, renal fibrosis

## Abstract

Precise regulation of cell cycle is essential for tissue homeostasis and development, while cell cycle dysregulation is associated with many human diseases including renal fibrosis, a common process of various chronic kidney diseases progressing to end-stage renal disease. Under normal physiological conditions, most of the renal cells are post-mitotic quiescent cells arrested in the G0 phase of cell cycle and renal cells turnover is very low. Injuries induced by toxins, hypoxia, and metabolic disorders can stimulate renal cells to enter the cell cycle, which is essential for kidney regeneration and renal function restoration. However, more severe or repeated injuries will lead to maladaptive repair, manifesting as cell cycle arrest or overproliferation of renal cells, both of which are closely related to renal fibrosis. Thus, cell cycle dysregulation of renal cells is a potential therapeutic target for the treatment of renal fibrosis. In this review, we focus on cell cycle regulation of renal cells in healthy and diseased kidney, discussing the role of cell cycle dysregulation of renal cells in renal fibrosis. Better understanding of the function of cell cycle dysregulation in renal fibrosis is essential for the development of therapeutics to halt renal fibrosis progression or promote regression.

## Introduction

Tight regulation of cell cycle is essential for mammalian tissue homeostasis and development, whereas cell cycle dysregulation leads to many human diseases such as cancer, cardiovascular disease, inflammation, and neurodegenerative diseases (Wiman and Zhivotovsky, 2017). Renal fibrosis is a common process of almost all chronic kidney diseases (CKDs) progressing to end-stage renal disease (ESRD). More than a decade of studies have found that cell cycle dysregulation of the renal tubular epithelial cells (TECs) could promote injured kidneys caused by toxins, hypoxia, and metabolic disorders to progress to CKD (Susnik et al., 2015; Moonen et al., 2018).

Under normal physiological conditions, adult mammalian renal cell turnover is very low; most of the renal cells are arrested in G0 phase of the cell cycle (Thomasova and Anders, 2015). Injuries, such as ischemic, toxic, and obstructive injuries, could promote the activation of cell cycle and initial cell proliferation of renal cells, which is an important compensatory mechanism to restore renal function. Mild injuries could be repaired through cell proliferation of renal cells; therefore, renal function could be fully recovered and most renal cells re-enter the G0 phase. However, when the injury is more severe or repeated, cell cycle of renal cells is dysregulated, manifesting as cell cycle arrest or overproliferation, both of which are closely related to renal fibrosis (Canaud and Bonventre, 2015; Yan et al., 2016).

To address the complex needs of kidney to keep homeostasis and repair, a delicate system to regulate cell cycle progression of renal cells is needed, that is, cell cycle control system (Harashima et al., 2013; Pack et al., 2019). However, this system could be disturbed by severe or repeated injuries, leading to cell cycle dysregulation and renal fibrosis. Recent works have improved our understanding of how cell cycle dysregulation of renal cells regulates the progression of renal fibrosis (Lovisa et al., 2015; Li et al., 2016; Liu et al., 2019b; Liu T. et al., 2019; Koyano et al., 2019; Zhao et al., 2020; Hanai et al., 2021). In this review, we focus on cell cycle regulation of renal cells in mammalian healthy and fibrotic kidney, discussing the relationships between cell cycle dysregulation of different renal cells and renal fibrosis, and finally putting some open problems about cell cycle modulation in renal fibrosis. The better understanding of the function of cell cycle dysregulation of renal cells in renal fibrosis is essential for developing strategies to halt or reverse renal fibrosis progression.

## Features of the Mammalian Cell Cycle

**FIGURE 1:**
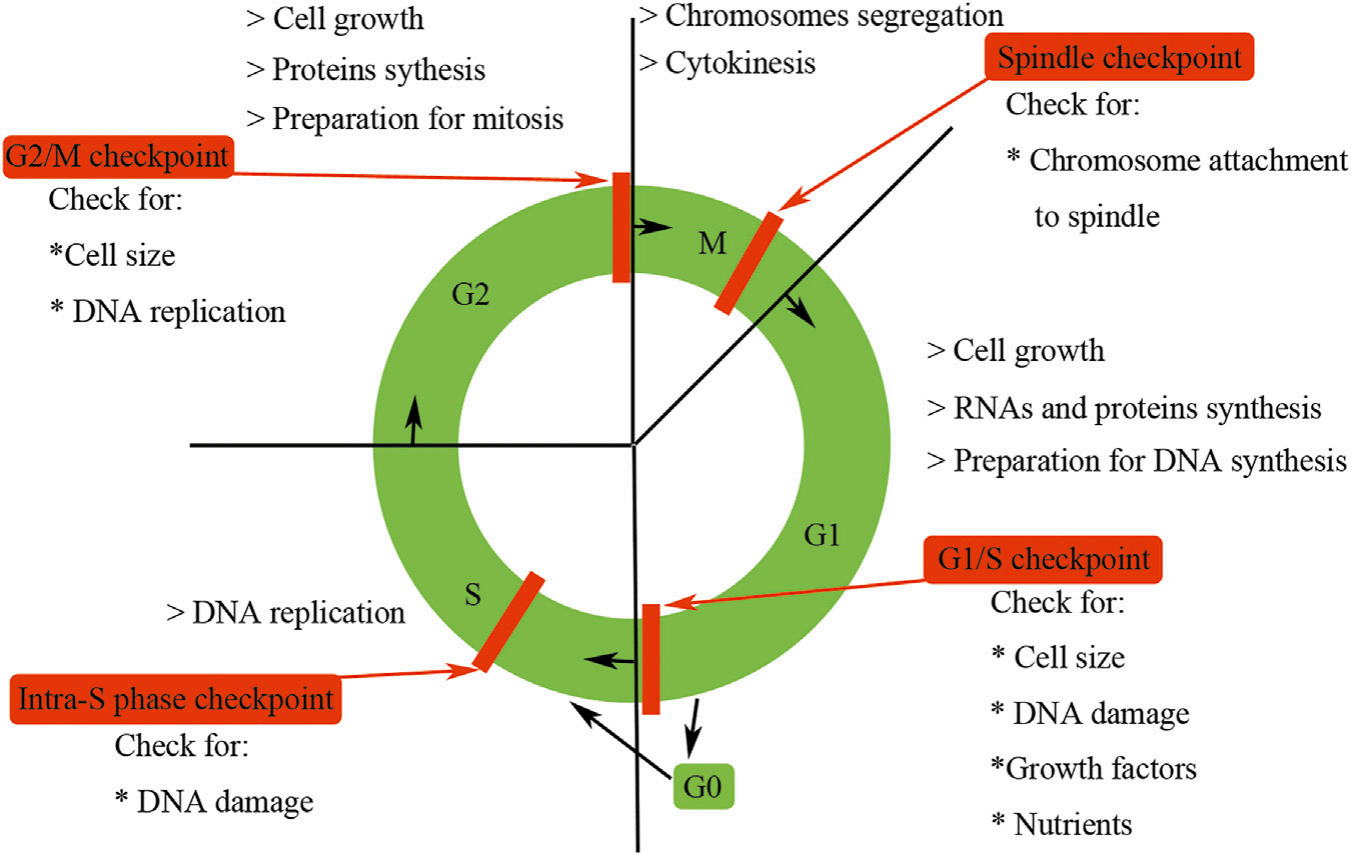
Features of the mammalian cell cycle. Mammalian cell cycle is tightly regulated and can be artificially divided into four distinct phases (G1, S, G2, and M) according to their specific characteristics. G0 phase is usually used to describe cells that have exited the cell cycle and become quiescent. The progression of the mammalian cell cycle is precisely controlled by CDKs, cyclins, and CKIs. Checkpoints could ensure the processes at each phase of the cell cycle have been accurately completed before entering into the next phase.

Cell cycle begins from the completion of one division to the end of the next, leading to the generation of two daughter cells. Mammalian cell cycle is tightly regulated and can be artificially divided into four distinct phases (G1, S, G2, and M) according to their specific characteristics (Liu et al., 2019a; Martínez-Alonso and Malumbres, 2020) (Figure 1). G1 phase is the gap phase, which is characterized by cell growth in size and the synthesis of RNAs and proteins required for DNA duplication. S phase is the synthesis phase during which DNA is synthesized. G2 phase is another gap phase, in which stage cells are characterized by rapid growth in cell size, more protein synthesis and preparation for division. M phase is the mitosis phase, during which the replicated chromosomes are segregated into separate nuclei and cytokinesis promoting the formation of two daughter cells. At the end of the M phase, 1 cell divides into two daughter cells, each of which contains one copy genomic DNA of the mother cell, and a cell cycle is accomplished.

Besides G1, S, G2, and M phases, the term G0 phase is usually used to describe cells that have exited the cell cycle and become quiescent. For example, under normal physiological conditions, most of the mammalian renal cells are arrested in G0 phase. However, Vogetseder et al. have found that a large number of rat epithelial cells in the proximal tubule were not in G0 phase but in G1 phase of the cell cycle (Vogetseder et al., 2008; Witzgall, 2008). Cells in G0 phase could be activated by internal or external stimuli and then re-enter the G1 phase. Some highly differentiated cells, such as neurons or cardiomyocytes, need to exit from the cell cycle permanently so as to satisfy the demands of functional requirements.

The progression of the mammalian cell cycle is tightly regulated by cyclin-dependent kinases (CDKs), cyclins, and cyclin-dependent kinase inhibitors (CKIs) (Morgan, 1997). CDKs drive the events of the mammalian cell cycle and control the rhythm of mammalian cell cycle procession; besides, they also integrate extracellular and intracellular signals to ensure the fine coordination of cell cycle events (Morgan, 1997). CDKs function as cell cycle event drivers, which are completely dependent on the association with cyclins, being first found in sea urchin eggs by their cyclic oscillations during the cleavage division in the early 1980s (Evans et al., 1983). Oscillating synthesis of cyclins controls the stage-specific timing of CDK activity. The association of cyclins is the primary determinant of CDK activity. Besides cyclins, other additional regulatory subunits—CKIs—are needed to modulate CDK activity, substrate recognition, and subcellular location. CDKs, cyclins, and CKIs form a finely tuned regulatory network to ensure precise progression of the cell cycle. Besides their well-established function in cell cycle control, increasing studies have found that mammalian cell cycle regulators also play an essential role in other biological processes such as transcription, epigenetic regulation, metabolism, stem cell self-renewal, neuronal functions, and spermatogenesis (Lim and Kaldis, 2013).

To ensure genomic integrity and the faithful transmission of correct replicated DNA during cell division, mammals have evolved a quality control system called checkpoint, which presents in different phases of the cell cycle (Johnson and Walker, 1999) (Figure 1). The presence of these checkpoints ensures that the processes at each phase of the cell cycle are accurately completed before entering the next phase. The first checkpoint of the mammalian cell cycle is the G1/S checkpoint, which checks for cell size, nutrients, growth factors, and DNA damage, suspending cell cycle for DNA repair and maintaining the integrity of the genome (Johnson and Walker, 1999). The next checkpoint is the intra-S phase checkpoint, which can be activated by the DNA damage escaping from the G1/S checkpoint or occurring during the S phase, and halts the cell cycle in S phase (Johnson and Walker, 1999). The third checkpoint is G2/M checkpoint, which determines whether or not the cell continues to complete mitosis. Specifically, G2/M checkpoint ensures three important things: DNA has been well replicated, all replication errors have been rectified, and the cell size is big enough to divide (Johnson and Walker, 1999). The final checkpoint is the metaphase or spindle checkpoint, which ensures that the chromosomes have been well aligned on the spindle and are sufficient for mitosis (Johnson and Walker, 1999). These four checkpoints to some degree are redundant, but each of them has somehow relative specificity. Checkpoints are activated by incomplete DNA replication due to stalled replication forks, and damaged DNA induced by both internal and external sources such as UV light, ionizing radiation, reactive oxygen species, or DNA-damaging chemotherapeutic agents (Reinhardt and Yaffe, 2009). Checkpoint activation prevents further cell cycle progression of the damaged cells. Besides implementing cell cycle arrest, checkpoint signaling also triggers DNA repair pathways. If the DNA damage exceeds repair capacity, additional signaling cascades are triggered to eliminate these impaired cells.

## Cell Cycle Regulation after DNA Damage

**FIGURE 2:**
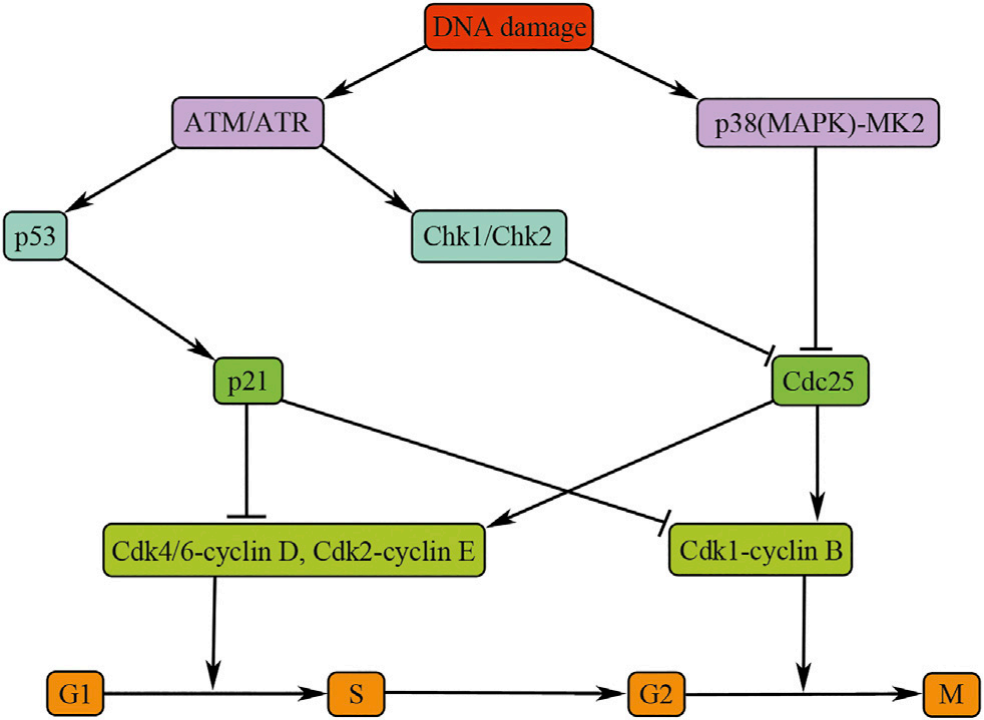
Cell cycle regulation after DNA damage. The figure illustrates the pathways and molecules that regulate the cell cycle upon DNA damage. Inhibition of Cdk4/6-cyclin D and CDK2-cyclin E is essential for G1/S arrest and blocking CDK1-cyclin B is necessary for G2/M arrest. ATM, ataxia telangiectasia mutated; ATR, ataxia telangiectasia and Rad3-related; Chk, checkpoint kinase; Cdc25, cell division cycle 25; CDK, cyclin-dependent kinase.

After DNA damage, mammalian cells will activate two major canonical kinase signaling pathways, that is, ataxia telangiectasia mutated/checkpoint kinase 2 (ATM/Chk2) and Rad3-related protein/checkpoint kinase1 (ATR/Chk1) signaling, to impede mammalian cell cycle progression and start DNA repair (Reinhardt and Yaffe, 2009). The ATM/Chk2 complex is activated by the DNA double-strand fracture, whereas the ATR/Chk1 pathway is activated primarily by DNA single-strand breaks. The ATM/ATR kinases regulate the G1/S, intra-S, and G2/M checkpoints by activating their downstream effector checkpoint kinases Chk2 and Chk1, respectively (Reinhardt and Yaffe, 2009). ATM/ATR could also phosphate p53 (Figure 2). In mammalian cells, p53-dependent signaling regulates G1/S arrest mainly through upregulation of p21 expression (Figure 2). p21 blocks cell cycle progression by inhibiting the Cdk2/cyclin E complex and therefore inhibiting the dissociation of Rb protein with transcription factor E2F (Vogelstein et al., 2000). Moreover, p21 can also inhibit the cell cycle progression at G2/M phase by the G2/M checkpoint after γ-irradiation or transforming growth factor beta (TGF-β) stimulation in renal epithelial cells (Bunz et al., 1998; Wu et al., 2013). In addition, p53-dependent pathway can also promote the impaired cells to initiate cell death program when the DNA damage is consistently accumulated (Vousden and Lu, 2002).

Both the ATM/Chk2 and the ATR/Chk1 pathways play their roles mainly through inactivating Cdc25 phosphatases, the positive regulators of cell cycle progression. The p38 (MAPK)/MK2 is a novel cell cycle checkpoint kinase pathway that integrates total stress responses with DNA damage (Reinhardt and Yaffe, 2009). This pathway responds to various intracellular and extracellular stimuli, including cytokines, hyperosmolarity, and UV irradiation and halts the progression of cell cycle in G2/M phase by inactivating Cdc25 (Roux and Blenis, 2004) (Figure 2).

## Cell Cycle Dysregulation in Kidney Fibrosis

Renal fibrosis is a common process of almost all CKDs progressing to ESRD and is a failure of wound healing process initiated by all kinds of injuries, such as toxins, hypoxia, and metabolic disorders (Liu, 2006; Wynn, 2008). Wound healing process is an evolutionary conserved defense program by which the injured tissue could be repaired and recovered. Leukocyte recruitment, angiogenesis, vascular leak, and the appearance of myofibroblasts are all involved in this process (Gabbiani and Majno, 1972). Originally, myofibroblasts are believed to be beneficial for the wound healing process, but more severe or consistently persistent injury leads to persistent presence of leukocytes and myofibroblasts, causing the maladaptive repair and finally resulting in tissue fibrosis (Dulauroy et al., 2012). Renal fibrosis is characterized by excessive deposition of extracellular matrix, which disrupts and replaces the functional parenchyma leading to organ failure. Besides, it affects all three main compartments of kidney, glomerulosclerosis in glomeruli, interstitial fibrosis in tubulointerstitium, and arteriosclerosis and perivascular fibrosis in vasculature (Djudjaj and Boor, 2019). During the process of renal fibrosis, almost all the cell types in the kidney, including fibroblasts, tubular epithelial cells, mesangial cells, and podocytes, are involved in this process, suggesting that it is a very complicated process (Boor et al., 2010; Zeisberg and Neilson, 2010). An increasing body of evidences have suggested that cell cycle dysregulation of these renal cells is closely related to renal fibrosis, especially the TECs (Canaud and Bonventre, 2015; Thomasova and Anders, 2015). In ischemic, toxic, and obstructive mice models of acute kidney injury (AKI), Yang et al. first found that cell cycle G2/M arrest of TECs could induce renal fibrosis through promoting profibrotic cytokine production by TECs (Susnik et al., 2015). They also found that administration p53 inhibitor or removal of the contralateral kidney could promote TECs to bypass the G2/M arrest, alleviating renal fibrosis in the unilateral ischemic injured kidney, suggesting cell cycle dysregulation of renal cells is indeed involved in renal fibrosis. Further studies showed that TECs in the G2-M phase formed a special structure, target of rapamycin (TOR)–autophagy spatial coupling compartments (TASCCs), which could promote the production and secretion of profibrotic cytokines (Canaud et al., 2019).

### Cell Cycle Dysregulation of Podocytes and Renal Fibrosis

Podocytes are the highly specialized cells whose foot processes cover the basement membrane of the glomerulus and comprise the filtration slit diaphragms, therefore regulating blood filtration (Pavenstadt et al., 2003). Most human chronic kidney diseases exist with podocyte injury or podocyte loss (Mundel and Shankland, 2002; Nagata, 2016). The loss of podocytes and the inability to renew a damaged glomerulus with functional podocytes will ultimately result in glomerulosclerosis or scarring of the glomerulus (Barisoni et al., 1999). Diabetes and other systemic disease states can lead to podocyte injury and loss, which in turn results in ESRD (Kriz, 2002; Welsh et al., 2010).

Podocytes express cyclin A, cyclin B1, and cyclin D1 and CDK inhibitors (such as p21, p27, and p57). In the early stage of kidney development, Ki-67, which is a marker of the proliferated cells, was highly expressed in immature podocytes, whereas cyclin D1 and CKIs were dramatically downregulated; in the capillary loop stage, CKIs and cyclin D1 were intensely increased, whereas Ki-67, cyclin A, and cyclin B1 were not detectable (Nagata et al., 1998; Barisoni et al., 2000b). The expression changes of cyclins, CDKs, and CKIs were associated with podocytes exiting the cell cycle and differentiate into mature podocytes expressing the podocyte markers, such as WT-1 or podocalyxin (Nagata et al., 1998; Barisoni et al., 2000b). Under normal physiological conditions, mature podocytes are arrested in G0 quiescent phase and express high levels of CDK inhibitors. The constitutive and intense production of CKIs is necessary to maintain the function of the differentiated quiescent podocytes (Nagata et al., 1998). The high level of CKIs probably leads to mature podocytes lacking the ability to renew during adult life.

Severe injuries induce cell death and promote the proliferation of survival cells so as to compensate for the cell loss. As postmitotic and quiescent cells, podocytes do not readily proliferate after injuries; however, in some diseased situations, such as collapsing focal segmental glomerulosclerosis (FSGS), the podocytes were stained positive for proliferating cellular markers and some podocytes even existed as binuclear (Barisoni et al., 1999). Cyclins, CDKs, and CKI expression were also changed in collapsing FSGS and human immunodeficiency virus-associated nephropathy (HIVAN); in these diseased situations, p27, p57, and cyclin D disappeared in podocytes, whereas the p21, cyclin A, and Ki-67 were highly expressed (Barisoni et al., 2000a; Barisoni et al., 2000b; Shankland et al., 2000). These podocytes bypassed cell cycle restriction points and entered the cell cycle, but they were unable to complete cell cycle and finally causing podocytes loss via podocyte mitosis (mitotic catastrophe) (Liapis et al., 2013). In the setting of adriamycin-induced podocyte injury, the presence of p21 has a protective effect on the podocytes in this model of toxic podocytopathy (Marshall et al., 2010). In other glomerular diseases, such as membranous nephropathy, immune-mediated injury led to cyclin A and Cdk2 upregulation in podocytes, mitosis entry, and DNA synthesis. Although these podocytes entered mitosis, they were unable to successfully complete it, and podocytes manifested as multinucleated and absence of cytoplasmic division (cytokinesis).

Diabetic nephropathy is characterized by podocyte hypertrophy. In various experimental models of diabetic nephropathy, such as Zucker diabetic rats and db/db mice, both models of type II diabetes, or type I model, induced by streptozotocin administration, the increasing expression of p27 and p21 was identified (Kuan et al., 1998; Hoshi et al., 2002; Baba et al., 2005). Although diabetic p21 or p27 knockout mice were protected from glomerular hypertrophy and the development of progressive renal failure, the specific mechanism of podocyte hypertrophy and its role in renal fibrosis remain unknown (Kuan et al., 1998; Awazu et al., 2003). As the terminal consequence of podocyte injury, glomerulosclerosis is characterized by segmental obliteration of glomerular capillaries with the extracellular matrix and has been believed to be a process to the complete sclerosis without regression (D’Agati, 2012). As a typical feature of kidney disease, proteinuria is induced by the podocyte injury because slit membrane molecules, the actin cytoskeleton, and cell adhesion molecules have formed a tight network so as to maintain filtration barrier function, and defection of these components leads to proteinuria (D'Agati et al., 2011). Persistent or severe podocyte injuries lead to cell detachment, which is probably caused by mitotic catastrophe (Kriz and Lehir, 2005). As normal function of podocytes requires specific arrangement of the cellular actin cytoskeleton, this may lead to podocytes unable to further form the actin contractile ring required by cytokinesis, resulting in mature podocytes that are unable to complete cytokinesis. Binucleated podocytes are frequently seen in the urine, suggesting podocyte loss caused by mitotic catastrophe is involved in podocyte detachment (Liapis et al., 2013). Moreover, podocyte loss in adriamycin-induced nephropathy was alleviated through administration of an inhibitor of p53-dependent cell cycle arrest, MDM-2, further strengthening the hypothesis (Mulay et al., 2013).

**FIGURE 3:**
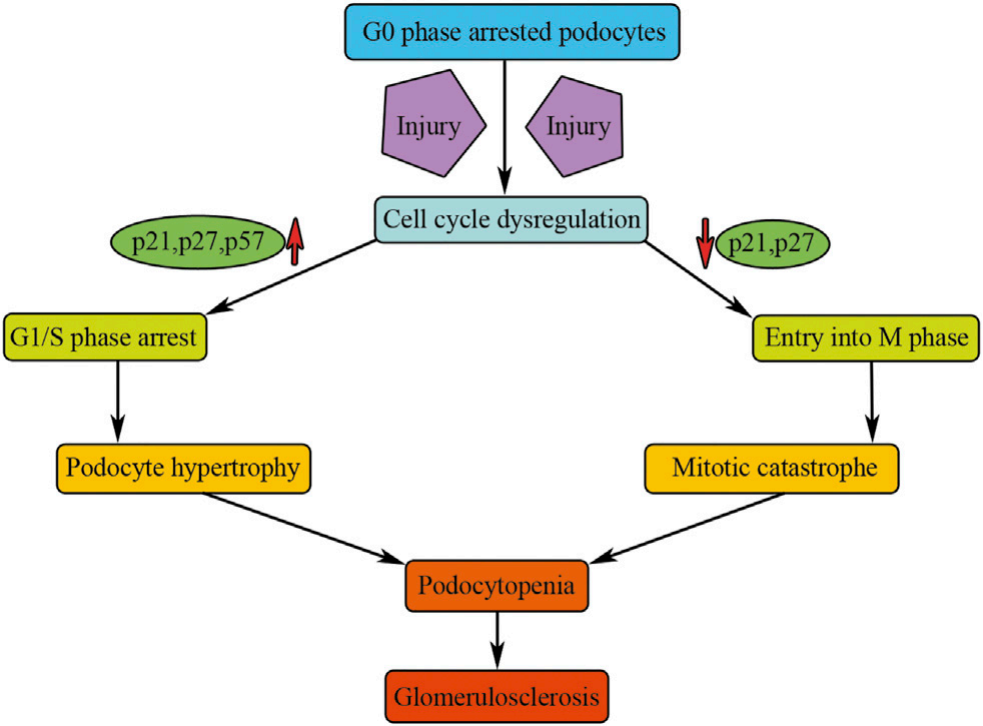
Cell cycle dysregulation of podocytes causes renal fibrosis. Injury could activate podocyte cell cycle entry and results in cell cycle dysregulation of podocytes, causing podocyte hypertrophy or mitotic catastrophe, which will result in podocytopenia and finally result in glomerulosclerosis.

In summary, under normal physiological conditions, mammalian podocytes are arrested in G0 quiescent phase and express high levels of CDK inhibitors. Injuries can induce podocyte death, whereas the remaining podocytes are unable to undergo regenerative proliferation to compensate for the loss of podocytes. Although podocytes can enter the cell cycle and can even undergo nuclear division in a variety of glomerular diseases, they were unable to complete normal cell division. The expression of CKIs, such as p21, p27, and p57, could lead to podocyte G1/S phase arrest, causing the abundant podocyte hypertrophy seen in progressive renal failure (Figure 3). When mature podocytes are forced to override cell cycle restriction point, they fulfill an aberrant mitosis followed by detachment and death through mitotic catastrophe (Figure 3). Such podocytes appeared multinucleated with aberrant mitotic spindles or micronuclei and were often found in several human and experimental glomerular diseases, such as HIVAN, FSGS, minimal change disease, immunoglobulin A (IgA) nephropathy, or adriamycin-induced nephropathy (Liapis et al., 2013; Mulay et al., 2013); all of these diseases exist with different degree of renal fibrosis.

### Cell Cycle Dysregulation of Renal Tubular Epithelial Cells and Renal Fibrosis

Under normal physiological conditions, mammalian mature TECs proliferate at a very low rate, which could be proved by PCNA and Ki-67 immunostaining (Nadasdy et al., 1994; Witzgall et al., 1994). Through this low rate proliferation, kidney can remedy the loss of TECs into the urine, which is few under normal conditions, probably one TEC per human nephron daily (Prescott, 1966). However, the rate of dividing cells remarkably increases after AKI so as to remedy TEC loss (Humphreys et al., 2008). If the injury is mild, the surviving TECs could cover the exposed basal membrane and restore cell number by proliferation (Prescott, 1966; Witzgall et al., 1994; Lin et al., 2005). In addition to proliferation, surviving TECs can also differentiate and express the embryologic markers such as vimentin (Witzgall et al., 1994; Lin et al., 2005), and then redifferentiate into specialized TECs resulting in the recovery of the nephron (Humphreys et al., 2011).

**FIGURE 4:**
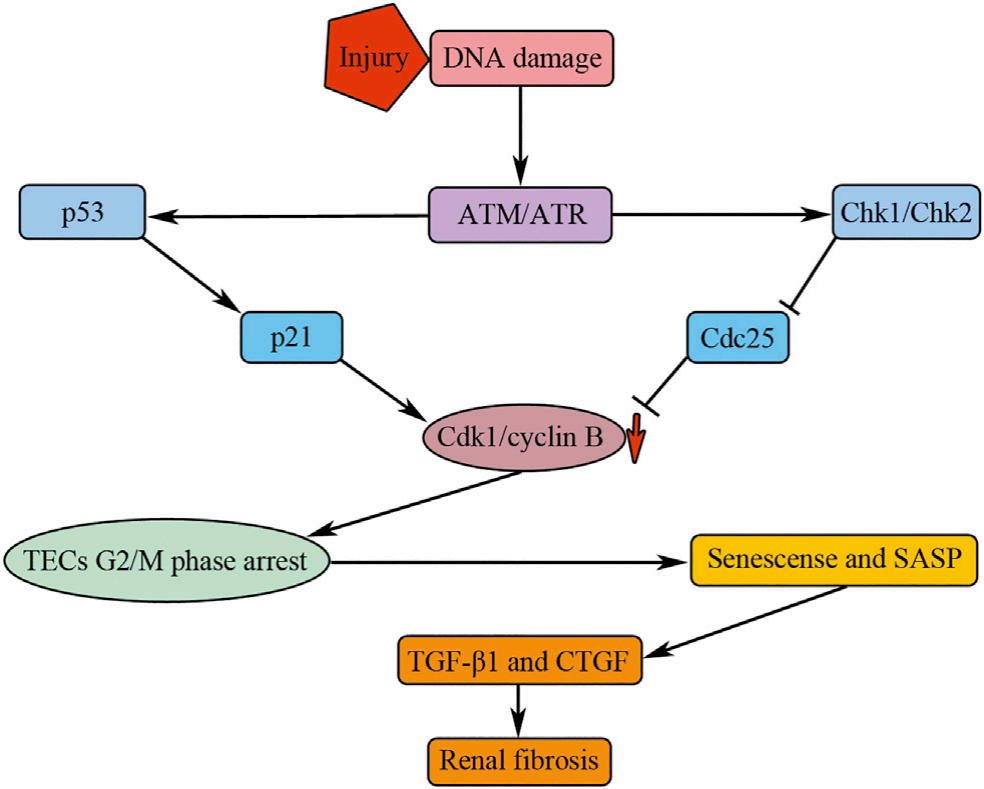
G2/M phase arrest of TECs mediates renal fibrosis by secreting profibrotic cytokines. Injuries result in the activation of ATM/ATR, which could promote the activation of Chk1/Chk2 and p53. p53 and Chk1/Chk2 could induce downregulation of Cdk1/cyclin B kinase activity by affecting p21 and Cdc25, respectively. The downregulation of Cdk1/cyclin B kinase activity results in TECs G2/M phase arrest; the arrested cells undergo senescence and manifest senescence-associated secretory phenotype (SASP), causing renal fibrosis by secreting profibrotic cytokines, such as TGF-β1 and CTGF.

However, when the damage is more severe or repeated, the repair process can be maladaptive, which will lead to incomplete structural and functional recovery of kidney tissue with persistent inflammation, activation, and proliferation of myofibroblasts, vascular rarefaction, increased production of interstitial matrix, and finally resulting in the progression of fibrosis (Grgic et al., 2012). Upon severe injuries, some TECs will arrest in the G2/M phase of cell cycle and mediate renal fibrosis by secreting profibrotic cytokines such as CTGF and TGF-β1 (Yang et al., 2010; Cosentino et al., 2013; Tang et al., 2013; Zhao et al., 2020) (Figure 4). Drug intervention causing an increasing TECs G2/M phase arrest after AKI could aggravate kidney fibrosis, whereas interventions which reduce TECs G2/M phase arrest result in less renal fibrosis (Yang et al., 2010; Cosentino et al., 2013; Gasparitsch et al., 2013; Tang et al., 2013; Zhao et al., 2020). Hence, TECs G2/M phase arrest properly is a novel histologic biomarker of renal fibrosis.

Many factors could influence TECs G2/M phase arrest and therefore affect renal fibrosis outcome. During the process of renal fibrosis, TECs undergo a partial EMT program; during this process, TECs still keep associated with their basement membrane but can express cellular markers of both epithelial and mesenchymal cells (Lovisa et al., 2015). During fibrotic injury, the partial EMT program led to a TEC G2/M phase arrest of the cell cycle; inhibition of partial EMT program can alleviate TEC G2/M phase arrest and attenuate interstitial fibrosis (Lovisa et al., 2015). Specific knockout of Atg5 gene in mouse TECs can destroy TEC autophagy and aggravate the TEC G2/M phase arrest, leading to aggravation of renal fibrosis upon kidney injury (Li et al., 2016). Specific knockout Numb in mouse TECs can alleviate TEC G2/M phase arrest and renal fibrosis induced by unilateral ureteral obstruction or unilateral ischemic renal injury (Zhu et al., 2016). Conventional knockout of Cyclin G1 can alleviate TEC G2/M phase arrest and renal fibrosis induced by severe kidney injury in mice (Canaud et al., 2019). Inhibition phosphorylation of 4E-BP1, a downstream effector molecule of mTORC1 pathway, can alleviate the TECs G2/M phase arrest and renal fibrosis (Sun et al., 2019). Therefore, TECs G2/M phase arrest is a common characteristic of renal fibrosis induced by various injures; however, the specific mechanism causing TECs G2/M phase arrest is still unclear.

Cell cycle regulators probably involve in the process of TECs G2/M phase arrest. Injuries can induce DNA damage of TECs, causing the activation of ATM/ATR (Kishi et al., 2019), which can further activate their downstream target genes Chk1/Chk2 and p53 (Reinhardt and Yaffe, 2009). Chk1/Chk2 inhibits Cdc25, the activator of Cdk1/cyclin B, causing G2/M phase arrest by downregulating the Cdk1/cyclin B kinase activity (Figure 4). p53 could activate its downstream target gene p21, causing G2/M phase arrest by downregulating the Cdk1/cyclin B kinase activity (Vogelstein et al., 2000) (Figure 4). Roscovitine, an inhibitor of Cdks, have been found to have the anti-fibrosis function (Steinman et al., 2012). In high glucose cultured HK-2 cells, a human proximal renal tubular epithelial cell line, roscovitine, can successfully reduce α-SMA expression and increase E-cadherin expression, suggesting that it can inhibit the EMT process of TECs (Wang et al., 2019). Further studies showed that roscovitine inhibited TECs EMT by inhibiting the upregulation of TGF-β1/p38MAPK pathway in HK-2 cells cultured with high glucose (Wang et al., 2019). In diabetic mice, administration of roscovitine can remarkably alleviate renal functional and histological injuries through inhibiting the expression of collagen, α-SMA, and TGF-β1 (Wang et al., 2019).

Consequently, although the TECs do not abundantly transdifferentiate into myofibroblasts, the G2/M phase arrested TECs could mediate renal fibrosis through paracrine pathway that is reinforced by a state of senescence characterized by the production of profibrotic cellular factors (Canaud et al., 2019) (Figure 4). In addition, p21 overexpression could induce the senescence of TECs which is involved in early stage of diabetic nephropathy in streptozotocin-induced diabetes 1 model (Kitada et al., 2014). In contrast to protective effect of p21 in AKI, the continued p21 activation may result in renal fibrosis, as the p21 knockout mice did not develop chronic kidney failure after 5/6 nephrectomy (Megyesi et al., 1999). It has been suggested that deletion of p21 allows hyperplastic compensatory proliferation of residual kidney tissue and prevents maladaptive hypertrophy (Megyesi et al., 1999).

### Cell Cycle Dysregulation of Mesangial Cells and Renal Fibrosis

**FIGURE 5:**
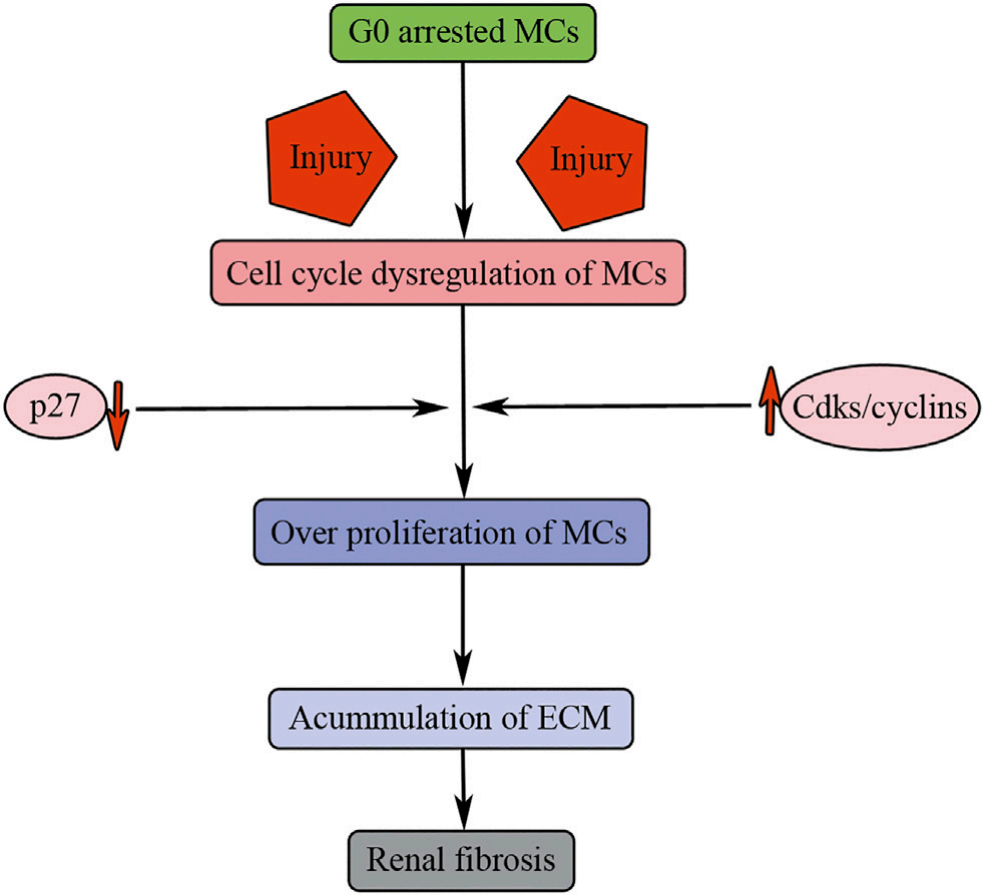
Cell cycle dysregulation of MCs results in renal fibrosis. Injuries result in G0 arrested MC entry into cell cycle. p27 downregulation and Cdks/cyclin upregulation induce overproliferation of MCs, accumulation of ECM, and finally causing renal fibrosis.

Mesangial cells (MCs) offer structural support for the glomerular tuft partially through the secretion and maintenance of the extracellular matrix. There is less MC proliferation in the adult mammalian healthy kidney, probably because under normal conditions MCs are either not exposed to mitogens or protected from them by inhibitory factors. Mature MCs remain in the G0 quiescent state by upregulation of the cell cycle inhibitor p27 (Combs et al., 1998). The initial of MC division could be stimulated by mitogen of injuries accompanied by a decrease in the expression of p27 (Figure 5).

In response to all kinds of injury stimulation, the quiescent MCs were stimulated to proliferation, resulting in an increasing number of MCs and persistent cellular matrix accumulation, and finally causing glomerulosclerosis. These characteristics could be found in diseases such as IgA nephropathy, lupus nephritis, membranoproliferative glomerulonephritis, and diabetic nephropathy. In the model of mesangial proliferative glomerulonephritis, Thy1 nephritis, the expression of cyclin D, cyclin E, cyclin A, CDK2, and CDK4 were increased during the phase of marked mesangial proliferation (Schöcklmann et al., 1999).

MC proliferation and the resulting matrix formation are the major characteristics of glomerular injury and fibrosis (Johnson, 1994; Shankland et al., 1996). Roscovitine, which can block the activity of CDK2, has been studied in experimental glomerulonephritis (Pippin et al., 1997b). It can decrease MC proliferation and the resulting matrix production (such as collagen type IV, laminin, and fibronectin), leading to alleviated renal fibrosis and improved renal function, suggesting that inhibiting MC overproliferation may be a useful therapeutic targeting for renal fibrosis (Pippin et al., 1997a). In the Thy1 model, accompanied by the onset of MC proliferation, the expression of p27 strikingly decreased. If experimental nephritis is induced in p27 knockout mice, the MC proliferation initial earlier and the proliferative response is bigger, accompanied by prominent extracellular matrix (ECM) accumulation (Marshall and Shankland, 2006). T-type calcium channels play an essential role in MC proliferation by targeting the G1/S checkpoint. Blocking of these channels by pharmacological drugs could inhibit MC proliferation by arresting them in G1 phase and alleviates glomerular damage in Thy1 model (Cove-Smith et al., 2013).

Statins, the cornerstone hormone drugs to manage dyslipidemia, have been found to have lipid-independent benefits against renal injury and fibrosis (Kostapanos et al., 2009; Chen et al., 2019). For example, statins can inhibit mesangial expansion, and the resulting extracellular matrix accumulation in the glomeruli of diabetic animal kidneys, and therefore attenuate renal fibrosis (KIM et al., 2000; Fujii et al., 2007). Moreover, *in vitro* studies had found that statins can inhibit the proliferation of cultured MC, which focal or diffuse proliferation is a typical characteristic of glomerular pathology (O'Donnell et al., 1993; Terada et al., 1998; Danesh et al., 2002). Lovastatin can dose-dependently inhibit DNA replication and proliferation of rat MCs, which can be reversible by added mevalonate (O'Donnell et al., 1993). Further study showed that the effect of lovastatin to inhibit MC proliferation through upregulation of a CDK inhibitor, p27Kip1, protein levels, as knockdown of p27Kip1 showed strikingly decreasing lovastatin-induced cell cycle arrest (Terada et al., 1998). Another study found that the proliferation of MCs induced by high glucose was accompanied by the decrease in p21 protein expression and the increase in CDK4 and CDK2 kinase activities. Simvastatin can increase p21 protein expression and downregulate CDK4 and CDK2 kinase activities (Danesh et al., 2002). These studies suggest that statins can inhibit renal injury and renal fibrosis independently of their cholesterol-lowering effect, such as inhibition of MC proliferation.

Therefore, MC overproliferation after injuries can contribute to renal fibrosis through persistent accumulation of ECM. Under normal physiological conditions, mammalian mature MCs are arrested in the G0 phase of cell cycle through upregulation of the CDK inhibitor p27 (Figure 5). However, upon injuries, the quiescent MCs are stimulated to overproliferation causing persistent cellular matrix accumulation, and finally causing renal fibrosis (Figure 5).

### Cell Cycle Dysregulation of Fibroblasts and Renal Fibrosis

Renal fibrosis is characterized by deposition of extracellular matrix in the potential space between tubules and peritubular capillaries. It is generally believed that myofibroblasts are the primary extracellular matrix-producing cells that produce a fair amount of interstitial matrix components, such as fibronectin and type I and type III collagens. Considering this, one of the key problems in the field is to study the origin of these matrix-producing myofibroblasts (Grande and Lopez-Novoa, 2009; Meran and Steadman, 2011; Schrimpf and Duffield, 2011).

It has been supposed that myofibroblasts have at least five different sources in mammalian fibrotic kidney, including activation of interstitial fibroblasts, differentiation of pericytes, translation of tubular epithelial cells and endothelial cells and recruitment of circulating fibrocytes (Barnes and Gorin, 2011). It has been believed that matrix-producing myofibroblasts mostly derive from resident fibroblasts through activation after kidney injury (Hewitson, 2009). Although this perception has recently been challenged, it is generally accurate (Strutz and Zeisberg, 2006; Grande and Lopez-Novoa, 2009). Recently, Kuppe et al. have found that distinct subpopulations of pericytes and fibroblasts were the main sources of myofibroblasts during human kidney fibrosis through the single-cell RNA sequencing technology (Kuppe et al., 2021). Moreover, they also showed that NKD2 may be a myofibroblast-specific target in human kidney fibrosis, as overexpression of NKD2 in human fibroblast cell line promoted the expression of ECM molecules, whereas knockout of NKD2 markedly downregulated the expression of ECM molecules (Kuppe et al., 2021). Blocking fibroblast to myofibroblast transformation can effectively inhibit renal fibrosis (Gerarduzzi et al., 2017; Li N. et al., 2020), suggesting that fibroblast is the major source of myofibroblast.

Under normal physiological conditions, renal fibroblasts are located in the interstitial space between the capillaries and the tubular epithelia and take shape a network in the whole renal parenchyma, so as to stabilize tissue structure (Kaissling and Le Hir, 2008). These cells are stellate shaped and contain abundant rough endoplasmic reticulum, collagen-secreting granules and actin filaments. They involve multiple cell processes, which keep them in contact with the tubular and capillary basement membranes (Kaissling and Le Hir, 2008). Under normal physiological conditions, renal fibroblasts stay in quiescent G0 phase of cell cycle and express CD73 (also known as ecto-5′-nucleotidase) in their plasma membrane and produce erythropoietin (Kaissling and Le Hir, 2008; Paliege et al., 2010). PDGFRβ and FSP1 were also expressed in fibroblasts (Floege et al., 2008; Grigorian et al., 2008; Boye and Mælandsmo, 2010; Boor and Floege, 2011). Fibroblasts control interstitial matrix physiological homeostasis by producing a few ECM components in normal conditions. However, after injury, fibroblasts are activated and acquiring the ability to proliferate and translating to myofibroblast that expressing α-SMA, generating a huge amount of ECM components. Myofibroblasts also retain FSP1 and PDGFRβ expression and express vimentin *de novo*.

**FIGURE 6:**
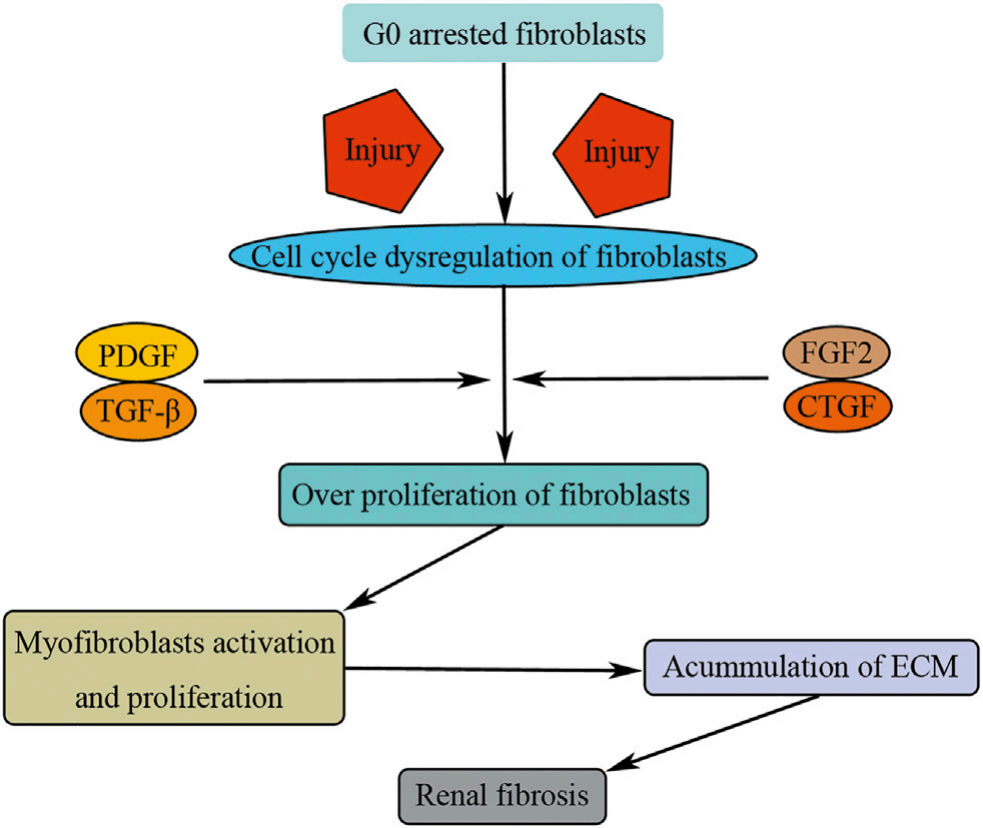
Cell cycle dysregulation of fibroblasts induces renal fibrosis. Injuries result in G0 arrested fibroblast activation and entry into cell cycle. Growth factors (PDGF, TGF-β, FGF2, and CTGF) promote fibroblast overproliferation and translate to myofibroblasts, causing renal fibrosis by consistent accumulation of ECM.

Activated fibroblasts possess two typical characteristics, that is, proliferation and myofibroblastic activation. The latter manifests as the expression of α-SMA and the production of extracellular matrix. Fibroblasts and myofibroblasts are overproliferated under the stimulus of cytokines, which results in the increasing number of myofibroblasts and accumulation of ECM in injured kidney. Growth factors such as PDGF, TGF-β, FGF2, and CTGF are well-known mitogens promoting fibroblast overproliferation (Strutz et al., 2000; Böttinger, 2007; Phanish et al., 2010; Ostendorf et al., 2012) (Figure 6). Besides these well-known cytokines, tissue-type plasminogen activator can also promote fibroblast overproliferation and myofibroblastic activation by recruitment of β1 integrin (Hu et al., 2007; Hu et al., 2008; Hao et al., 2010; Lin et al., 2010).

Therefore, fibroblasts are the main source of matrix-producing myofibroblasts. Under normal physiological condition, fibroblasts stay in quiescent state of G0 phase. Upon injuries, fibroblasts are activated and overproliferated, translating to myofibroblasts, causing renal fibrosis by consistent accumulation of ECM (Figure 6). However, the specific mechanism as to how the fibroblast is activated and overproliferated upon injuries remains unclear, and other types of renal cells, such as TECs, play an essential role during this process (Li X. et al., 2020). For example, tubule-derived exosomes can promote renal fibrosis through promoting fibroblast activation and proliferation (Liu et al., 2020).

## Conclusions and Perspectives

Mammalian cell cycle is tightly regulated by cell cycle regulators, such as CDKs, cyclins, and CKIs. These cell cycle regulators make sure that cell cycle is regulated precisely, which is essential for mammalian renal cell homeostasis and keeping normal renal function. However, severe or repeated injuries could induce dysregulation of cell cycle manifesting as cell cycle arrest or overproliferation, both of which are closely related to renal fibrosis.

**FIGURE 7:**
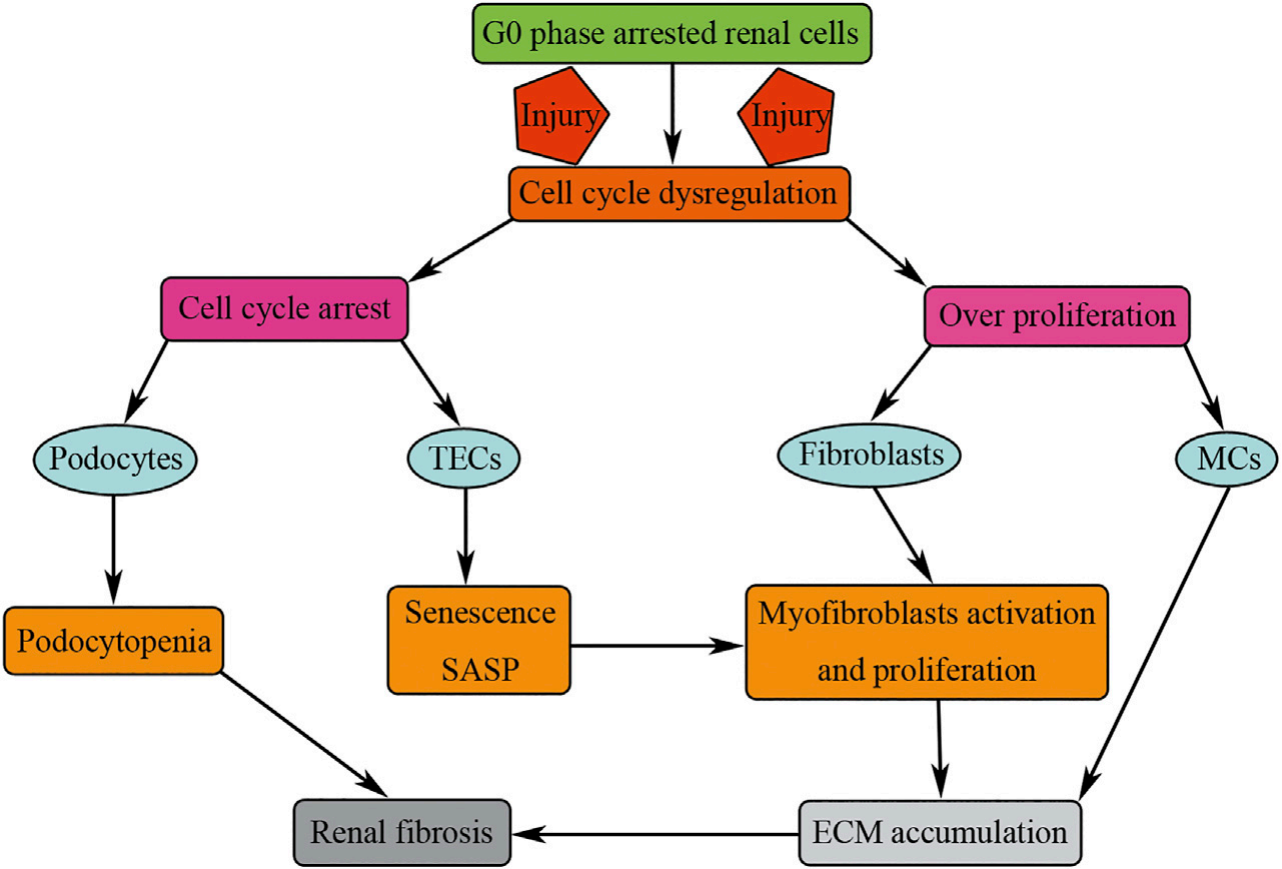
Cell cycle dysregulation of renal cells results in renal fibrosis. Injuries result in cell cycle dysregulation manifested as cell cycle arrest (podocytes and TECs) or overproliferation (fibroblasts and MCs). Cell cycle arrest or overproliferation of renal cells results in renal fibrosis by podocytopenia and consistent accumulation of ECM.

Under normal physiological conditions, most of the renal cells are quiescent cells, arresting in G0 stage; the turnover of renal cells is very low. On one hand, mild injuries could stimulate the proliferation of renal cells so as to compensate the renal cells loss and restore renal function; on the other hand, more severe or repeated injuries lead to cell cycle dysregulation of renal cells, promoting renal fibrosis (Figure 7). Cell cycle dysregulation of podocytes manifests as cell cycle entry but could not finish mitosis; cells may be arrested in M phase of cell cycle, causing podocyte loss through mitosis catastrophe, and finally resulting in renal fibrosis. Cell cycle dysregulation of TECs manifests as TECs G2/M phase arrest; the arrested cell undergoes senescence, promoting renal fibrosis by secreting profibrotic cytokines. Cell cycle dysregulation of MCs manifests as overproliferation resulting in persistent cellular matrix accumulation, and finally causing glomerulosclerosis. Cell cycle dysregulation of fibroblasts manifests as overproliferation and activation resulting in renal fibrosis by increasing the number of myofibroblasts and accumulation of ECM. Therefore, cell cycle dysregulation of renal cells may be a perfect target for the treatment of renal fibrosis.

Although cell cycle regulators play essential roles in renal fibrosis, very few cell cycle regulators have been researched in renal fibrosis. Indeed, most of the researched regulators were CKIs, such as p53 and its target protein p21. p21 was upregulated in the kidney after injury (Megyesi et al., 1996). Compared with wild-type mice, p21 knockout mice manifested as more severe kidney dysfunction, more severe kidney damage, and higher rate of mortality rate after AKI (Megyesi et al., 1998; Nowak et al., 2003). In contrast, p21 knockout mice had less histologic lesions after sub-total nephrectomy with enhanced tubular proliferation compared with wild-type mice (Megyesi et al., 1999). p53 was also found to be upregulated in the kidney after injury and its inhibition or gene deletion reduced kidney lesions and renal fibrosis (Wei et al., 2007; Molitoris et al., 2009; Yang et al., 2010; Zhou et al., 2010; Ying et al., 2014; Liu et al., 2019c; Tang et al., 2019). Besides the p53 and p21, there is very little information available about other cell cycle regulators in renal fibrosis. The precise role of each CDK, cyclin, and CKI in different renal cells during renal fibrosis deserves a good deal more investigation.

ACE inhibitors, statins, anticoagulants, glucocorticoids, cyclophosphamide, azathioprine, and mTOR inhibitors are the common medicines used in kidney diseases; most of them can affect cell proliferation in some way. However, the role of these medicines in cell cycle progression of specific renal cells is largely unknown. Statins can inhibit MC proliferation by suppression of the Rho and Ras pathway (Kostapanos et al., 2009). Rapamycin, the inhibitor of mTOR, can mitigate the hypertrophy in diabetes model through downregulation of p70S6 kinase pathway (Sakaguchi et al., 2006). ACE inhibitors can decrease abnormal division of renal progenitor cells by deactivation of NCAM+ and thus alleviate lesions of hyperplastic in podocytopathies; however, this drug can also promote regeneration of glomeruli by the transcription factor C/EBPδ (Benigni et al., 2011; Rizzo et al., 2016). Upon severe injuries, MCs and fibroblast overproliferation promote renal fibrosis; therefore, inhibiting MCs and fibroblast proliferation by drugs is beneficial for the prevention of renal fibrosis. However, these drugs can also inhibit TECs’ appropriate proliferation, therefore inhibiting the kidney function restoration. In other words, drugs that inhibit cell proliferation could be harmful for some type of renal cells and aggravate renal fibrosis. Therefore, more additional researches should be conducted to elucidate the function of these drugs in cell cycle progression of specific renal cells and in renal fibrosis.

In conclusion, recent progression in the pathophysiology of renal fibrosis has emphasized the important roles of cell cycle dysregulation of renal cells in renal fibrosis. Although there are a lot of questions to be clarified, these findings open new avenues to better understand, prevent, and slow down renal fibrosis.
